# Juvenile Hormone receptor Met is essential for ovarian maturation in the Desert Locust, *Schistocerca gregaria*

**DOI:** 10.1038/s41598-019-47253-x

**Published:** 2019-07-25

**Authors:** Marijke Gijbels, Cynthia Lenaerts, Jozef Vanden Broeck, Elisabeth Marchal

**Affiliations:** 1 Research group of Molecular Developmental Physiology and Signal Transduction, KU Leuven, Zoological Institute, Naamsestraat 59 box 2465, 3000 Leuven, Belgium; 2 Present Address: Imec, Kapeldreef 75, B- 3001 Leuven, Belgium

**Keywords:** RNAi, siRNAs

## Abstract

Juvenile hormones (JH) are key endocrine regulators produced by the corpora allata (CA) of insects. Together with ecdysteroids, as well as nutritional cues, JH coordinates different aspects of insect postembryonic development and reproduction. The function of the recently characterized JH receptor, Methoprene-tolerant (Met), appears to be conserved in different processes regulated by JH. However, its functional interactions with other hormonal signalling pathways seem highly dependent on the feeding habits and on the developmental and reproductive strategies employed by the insect species investigated. Here we report on the effects of RNA interference (RNAi) mediated *SgMet* knockdown during the first gonadotrophic cycle in female desert locusts (*Schistocerca gregaria*). This voracious, phytophagous pest species can form migrating swarms that devastate field crops and harvests in several of the world’s poorest countries. A better knowledge of the JH signalling pathway may contribute to the development of novel, more target-specific insecticides to combat this very harmful swarming pest. Using RNAi, we show that the JH receptor Met is essential for ovarian maturation, vitellogenesis and associated ecdysteroid biosynthesis in adult female *S. gregaria*. Interestingly, knockdown of *SgMet* also resulted in a significant decrease of *insulin-related peptide* (*SgIRP*) and increase of *neuroparsin* (*SgNP*) 3 and 4 transcript levels in the fat body, illustrating the existence of an intricate regulatory interplay between different hormonal factors. In addition, *SgMet* knockdown in females resulted in delayed display of copulation behaviour with virgin males, when compared with *dsGFP* injected control animals. Moreover, we observed an incapacity of adult *dsSgMet* injected female locusts to oviposit during the time of the experimental setup. As such, *SgMet* is an essential gene playing crucial roles in the endocrine communication necessary for successful reproduction of the desert locust.

## Introduction

The classic insect hormones, juvenile hormones (JH) and ecdysteroids, play critical roles in insect postembryonic development. In juvenile insects ecdysteroids trigger the moulting process, while JH will determine the nature of the moult^1^. These lipophilic hormones, together with nutritional cues, as well as signalling mechanisms directed by regulatory peptides, such as insulin-related peptides (IRP) and neuroparsins (NPs) also control the reproductive physiology of adult insects. However, the exact interplay between hormones and nutrition in this regulation may distinctly differ depending on the insect’s specific feeding habits and reproductive strategies^2,3^. The role of JH as a master regulating gonadotropin in insect female reproduction is established in phylogenetically basal species, such as orthopterans (the insect order to which locusts belong), while ecdysteroids seem to have taken over this leading role in some lepidopteran and most dipteran species^3,4^. In female locusts, JH, synthesized in the corpora allata (CA, part of the retrocerebral complex), stimulates vitellogenin (Vg) production in the fat body by activating *Vg* gene transcription^5^. Furthermore, JH also binds to membrane receptor sites in the ovarian follicular epithelium to initiate events that cause cells to shrink^6–10^. This process, called patency, allows Vgs, which are mainly produced by the fat body and then released into the haemolymph, to be taken up by the developing oocyte. Recent ligature experiments in both *Locusta migratoria* and *Schistocerca gregaria* have shown the presence of an oviduct derived Patency Inducing Factor (PIF) involved in the initiation of patency in the terminal follicle via the pedicel. The action of PIF, for which the chemical structure is currently unknown, is clearly boosted by JH^11^. In adult female locusts, ecdysteroids produced in the ovarian follicular cell layer appear to induce meiotic re-initiation in the oocyte^12^. In addition, they are incorporated as conjugates into the eggs where they act as a source of ecdysteroids during embryogenesis^13^. These conjugates can bind vitellins, thereby preventing their leakage into the haemolymph^13–15^. Furthermore, ecdysteroid signalling via the nuclear receptor complex consisting of the ecdysone receptor and the retinoid-X-receptor/ultraspiracle, plays a role in choriogenesis in adult female desert locusts^16^.

In contrast to the molecular basis of ecdysteroid action (as was reviewed by Roy *et al*.)^3^, the JH signalling pathway was only recently described with the characterization of the basic-helix-loop-helix (bHLH)/Per-Arnt-Sim (PAS) transcription factor, Methoprene-tolerant (Met). First described in *Drosophila melanogaster* as a factor involved in the resistance to the commercial Insect Growth Regulator (IGR) Methoprene^17^, the affinity binding study by Charles *et al*.^18^ ultimately confirmed the function of Met as JH receptor^18^. Upon JH binding, the Met-Met homodimer dissociates and becomes accessible to other bHLH-PAS proteins such as Taiman, a steroid receptor co-activator, which induces the expression of JH response genes^19,20^. RNA interference (RNAi) studies in the red flour beetle, *Tribolium castaneum*, the German cockroach, *Blattella germanica*, and the fire bug, *Pyrrhocoris apterus* have demonstrated the importance of Met and its downstream transcription factor krüppel-homolog 1 (Kr-h1) in the anti-metamorphic effects of JH^21–25^. Moreover, RNAi studies in multiple insect species have confirmed the conserved function of Met and Kr-h1 as key players in JH signalling in adults as well. The pleiotropic JH acts via Met and Kr-h1 to control different processes in adults, such as vitellogenesis in the fat body, lipid accumulation in the primary oocyte, regulation of mating and sex pheromone production^24,26–38^. Importantly, in another locust species, the migratory locust, *L. migratoria*, the molecular basis of JH action in reproduction is well established^20,39^. The RNAi-mediated knockdown of *Met* and *Kr-h1* prevents *Vg* expression in the fat body, ovarian development, lipid accumulation and patency in the oocyte. Several molecular players involved in fat body polyploidy, DNA replication and apoptosis, as well as in the folding of Vg proteins and patency have since then been identified^40–42^. However the interplay with other hormonal pathways and nutritional signalling in locust species remains unknown.

Our study focuses on the role of Met, the nuclear receptor that mediates JH signalling, in the female reproductive physiology of the desert locust, *S. gregaria*. The insulin signalling pathway (ISP), sensing nutrient status, plays a crucial role in the trade-off between reproduction and survival in insects, positively controlling vitellogenesis and oocyte growth^43,44^. Whether or not the ISP acts via a mediator action of ecdysteroids and JH remains ambiguous as evidenced by contradictory reports in different insect orders. Most likely, IRP, ecdysteroids and JH are involved in an intricate cross-talk, in which the insect’s nutritional status is a crucial determinant of this cross-talk’s output. For a more in depth summary on this topic, the reader is referred to Badisco *et al*.^45^, Van Wielendaele *et al*.^46^ and Roy *et al*.^3^. In the desert locust, *S. gregaria*, silencing *SgIRP* negatively affects *Vg* transcript levels and oocyte growth^47^. Since JH initiates vitellogenesis in this species, these earlier results from our lab suggest communication between both pathways. This same study showed that knockdown of the *S. gregaria NPs* (*SgNPs*) increases *SgVg* transcription, resulting in bigger oocytes. This finding was in line with the initial discovery of the first locust NP, a peptide purified from the pars intercerebralis-corpora cardiaca (CC) neurohaemal complex, which was shown to act as an anti-gonadotrophic factor, contrary to the effects of JH^48–50^. Locust NPs belong to a conserved family of arthropod peptides, but they were also reported to share sequence similarity with the binding region of vertebrate insulin-like growth factor binding proteins (IGFBP)^48^ and *Sg*NP4 was shown to be capable of interacting with *Sg*IRP *in vitro*^51^.

Desert locust swarms can threaten the livelihood of approximately one tenth of the world’s population by destroying agricultural production^52,53^. Unfortunately, the outbreak of such swarms often implies the use of classic insecticidal nerve toxins, resulting in a negative impact on the environment. In the fight against this pest it is therefore crucial to find targets that are specific enough to minimize effects on non-target organisms. Vital insect processes, such as metamorphosis and reproduction, are regulated by multiple hormonal pathways, which utilize molecular components that may prove to be excellent targets in the development of a next generation of more selective and eco-friendly insecticides. Using RNAi, we demonstrate that the JH receptor Met is crucial for the initiation of ovarian maturation and vitellogenesis, and that its key role is situated within a complex hormonal network involved in the control of reproduction in *S. gregaria*.

## Results and Discussion

### Description of the relative transcript levels of the genes of interest

**Figure 1:**
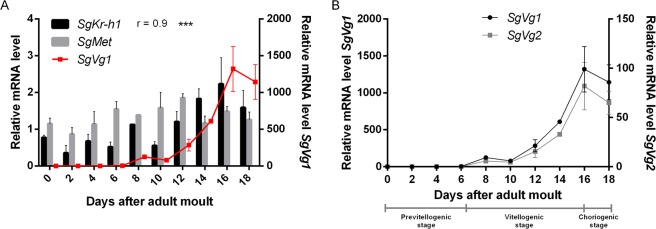
Relative transcript levels of *SgMet*, *SgKr-h1* and *SgVg*s in the fat body throughout the first gonadotrophic cycle. (**A**) *SgMet* + *SgKr-h1* and (B) *SgVg1* + *SgVg2* relative transcript levels were determined in the fat body of adult females throughout the first gonadotropic cycle (day 0 until day 18). (**B**) The different stages in which the first gonadotrophic cycle can be divided are included as well. *SgVg1* mRNA are given in **A**), similar temporal profiles for both *SgVg1* and *SgVg2* mRNA levels were observed as shown in **B**). The data represent mean ± S.E.M. of three independent pools of ten animals, run in duplicate and normalized to *β-actin* and *EF1α* transcript levels. The correlation coefficient (r) between the measurements was found via a Pearson correlation calculation. P-values are indicated by asterisks (***p < 0.001).

*SgMet* (accession number: MK855050) shows a wide tissue distribution profile. Its expression in 10-day-old adult locusts is mainly observed in the male accessory glands (AGs), CA, fat body, prothoracic glands (PGs) and (flight) muscles (Supplementary Fig. S1). *SgMet* transcript levels remain relatively stable in the fat body of adult female locusts throughout the first gonadotrophic cycle (Fig. 1A), a situation which is similar to what was reported on the expression of *Met* during the reproductive cycle of the pacific beetle cockroach, *Diploptera punctata* and the German cockroach, *B. germanica*^28,33^. Also the relative *Met* mRNA levels measured in the complete body of the cotton bollworm, *Helicoverpa armigera*, and the red flour beetle, *Tribolium castaneum*, remain stable during adult female development^36,38^. On the other hand, the temporal profile of the relative mRNA levels of the downstream transcription factor *SgKr-h1* correlate well with the transcript levels of two *Vg* genes (Fig. 1A). Since *Sg*Kr-h1 is a transcription factor acting directly downstream of the JH receptor Met, *SgKr-h1* transcript levels can be considered as a measure for Met-mediated JH signalling activity, as was previously described for *B. germanica*, *D. melanogaster* and *T. castaneum*^54–56^. The *SgVg* developmental profile shows a clear increasing trend which starts on day 6, when the basal oocytes enter their vitellogenic stage of maturation, and continues to increase until day 16, when these oocytes almost reach their maximal length. The population of animals from which these samples were taken, completed their first gonadotrophic cycle with oviposition around day 18 (Fig. 1C).

### Knockdown of the JH receptor Met affects expression of the downstream signalling component Kr-h1 and of JH biosynthetic enzymes

In order to verify the reproducibility of the results obtained, and to eliminate the possibility of off-target effects, two separate *dsSgMet* (*SgMet* dsRNA) constructs were designed and tested in the same experimental setup, which resulted in similar phenotypic observations.

**Figure 2:**
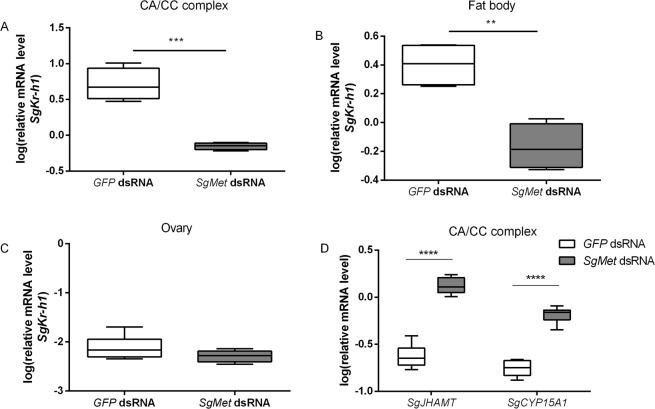
Relative transcript levels of *SgKr-h1* and two JH synthesis genes upon *dsSgMet* injections. Relative *SgKr-h1* transcript levels were measured in (**A**) the CA/CC complex, (**B**) the fat body and (**C**) the ovaries of 12-day-old *dsSgMet* or *dsGFP* injected adult female locusts. (**D**) Relative *SgJHAMT* and *SgCYP15A1* transcript levels were measured in the CA/CC complex as well. The data (log transformed) are shown as box plots (min to max) of four independent pools of five animals, run in duplicate and normalized to *β-actin* and *EF1α* transcript levels. Statistically significant differences (p) between the measurements were found, after a log transformation, via a t-test (with or without two-sided Welch’s correction) and are indicated by asterisks (**p < 0.01; ***p < 0.001****p < 0.0001).

The knockdown efficiency of the systemic RNAi of *SgMet* was assessed in different tissues on day 12 after adult eclosion (Supplementary Fig. S2). A significant downregulation was measured in the CA/CC complex (61%) and fat body (51%). However, *SgMet* levels were not downregulated in the ovaries. This can be explained by the previously described inefficient uptake of dsRNA into the follicle cells and oocytes of the desert locust^57,58^. In both the CA/CC complexes and the fat body, relative mRNA levels of *SgKr-h1* were shown to be significantly reduced with 88% and 73% respectively, upon silencing *SgMet* (Fig. 2A,B). Relative *SgKr-h1* mRNA levels were not affected in the ovaries as *SgMet* was not effectively silenced in this tissue (Fig. 2C). These findings confirm the transcription factor *SgKr-h1* to be situated downstream of *SgMet*.

The effect of silencing *SgMet* was also studied in the CA, the site of JH biosynthesis. Previous work already characterized the genes encoding the enzymes catalysing the penultimate and ultimate steps in the JH biosynthetic pathway of the desert locust^59^. Juvenile hormone acid methyl transferase (JHAMT) methylates farnesoic acid to methylfarnesoate (MF) and is known to act as the rate-limiting enzyme in JH biosynthesis^59–62^. The ultimate step in the pathway is catalysed by the cytochrome P450 enzyme CYP15A1 which epoxidizes MF to the active hormone, JHIII. Our current results show that silencing the JH receptor causes upregulation of the transcripts that code for the JH biosynthetic enzymes, *SgJHAMT* and *SgCYP15A1* (Fig. 2D), by 550% and 370%, respectively. Upon dissection of the CA, it also became apparent that silencing *SgMet* resulted in larger sized CA, again suggesting an increase in activity of these JH producing glands (Supplementary Fig. S3). Our data are in accordance with a recent study in the linden bug, *P. apterus*, where a rise in the circulating JH levels was observed upon silencing *Met*^24^. It can thus be hypothesized that in the CA *Sg*Met acts as a JH sensor involved in a negative feedback loop that keeps JH biosynthesis and CA activity under control, a homeostatic mechanism similar to what is often observed in endocrine gland regulation^63^.

### SgMet is crucial for initiating ovarian maturation, vitellogenesis and reproductive behaviour

**Figure 3:**
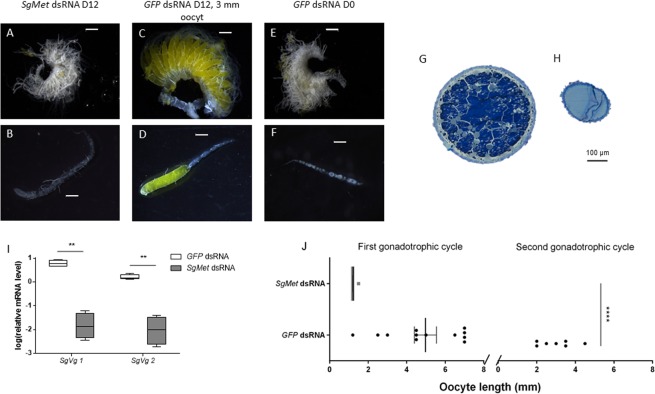
Arrest of ovarian maturation after silencing *SgMet*. Ovaries (**A**,**C**,**E**) and single ovarioles (**B**,**D**,**F**) of 12-day-old *dsSgMet* injected locusts (**A**,**B**), 12-day-old *dsGFP* injected (control) locusts (**C**,**D**) and freshly moulted (D0) control locusts (**E**,**F**). Scale bars: 1 mm. (**G**,**H**) Histological sections of 12-day-old (**G**) control and (**H**) *dsSgMet* injected locust oocytes. Scale bar: 100 µm. (**I**) Relative transcript levels of *SgVg1* and *SgVg2* in the fat body of 12-day-old *dsSgMet* injected and control female locusts. The data (log transformed) are shown as box plots (min to max) of four independent pools of five animals, run in duplicate and normalized to *β-actin* and *EF1α* transcript levels. Statistically significant differences (p) were found, after a log transformation, via a t-test (with or without two-sided Welch’s correction) and are indicated by asterisks (**p < 0.01). (**J**) The oocyte length (mm) of 12-day-old control and *dsSgMet* treated female locusts was observed. Each point represents the mean length of 3 randomly chosen oocytes (mean ± S.E.M). Statistically significant differences (p) were found via a nonparametric Mann-Whitney test and are indicated by asterisks (****p < 0.0001).

It should be noted that the locust reproductive cycle is strongly determined by environmental factors, which can cause variation in the exact timing. The first gonadotrophic cycle is therefore usually divided in different stages instead of exact time periods: (i) an early growth period during which the oocytes only reach a length of 0.8–0.9 mm; (ii) the previtellogenic stage, during which oocytes grow until they reach a length of 1.8 mm; (iii) the vitellogenic stage starting from 1.8 mm, characterized by patency, the shrinkage of the follicular epithelial cells, allowing passage of lipids and vitellogenin from the haemolymph into the developing oocytes, causing oocytes to grow until they reach about 7.5 mm; (iv) egg shell formation during the choriogenic stage and (v) ovulation and oviposition^64,65^. It is clear from Fig. 3J that the *SgMet* knockdown animals never enter the (pre)vitellogenic stage as the oocytes do not appear to have increased in length after 12 days in the adult stage when compared to newly moulted adult females. As such, it became evident that ovarian maturation was arrested by silencing *SgMet* (Fig. 3A–F). The ovaries of 12-day-old *dsSgMet* injected locusts remained arrested in a very immature, previtellogenic stage (Fig. 3A,B), still resembling the ovaries of freshly moulted control locusts (D0) (Fig. 3E,F). To further verify whether or not yolk materials were incorporated, transverse sections of oocytes were made (Fig. 3G,H). In oocytes of *dsGFP* injected control locusts, Vgs (blue matrix) as well as lipid droplets (greyish droplets) were visible. The accumulation of Vgs and lipid droplets was not observed in the *SgMet* knockdown animals. Moreover, when studying these sections, no patency of the follicular cell layer was observed even though higher JH levels might circulate in the haemolymph (due to the higher relative mRNA levels of *JHAMT* and *CYP15A1*; Fig. 2D). Our observations are similar to the lack of patency observed in both *L. migratoria* and *D. punctata* treated with *dsMet* which could not be rescued by treatment with methoprene^20,33^. Patency was long thought to be initiated by JH acting on membrane receptors in the ovary^6–10^. The recent report by Seidelmann *et al*.^11^ suggests that PIF, an as yet unidentified oviduct derived factor, is responsible for the initiation of patency in locusts and that its action is boosted by JH^11^. As no Vg was observable in oocytes of locusts where *SgMet* was knocked down, we analysed *SgVg* mRNA levels in fat body samples from *dsSgMet* versus *dsGFP* injected locusts on day 12 of the first gonadotrophic cycle (Fig. 3I). Silencing *SgMet* very obviously resulted in much lower relative *Vg* mRNA levels for both *SgVg1* and *SgVg2*, for which transcript levels during the first gonadotrophic cycle of wild type locusts are shown in Fig. 1C. In control knockdown animals, a large variation in basal oocyte lengths exists. Some of the control locusts were already in their second gonadotrophic cycle, characterised by orange lesions at the base of the new basal oocyte. In the *SgMet* knockdown locusts, it was evident that ovarian maturation was arrested. Oocytes longer than 1.5 mm were never observed, suggesting oocyte maturation could not be completed, even after 12 days in the adult stage (Fig. 3J). Moreover, dissection of the *SgMet* females at the end of the copulation experiment 22 days after adult eclosion revealed a continued arrest of oocyte development. It is apparent that JH-Met signalling is a common pathway regulating female vitellogenesis as evidenced by RNAi studies in different insect species, such as *T. castaneum*, *Colaphellus bowringi*, *Cimex lectularius*, *B. germanica*, *D. punctata*, *Aedes aegypti*, *H. armigera*, *R. prolixus*, *Reticulitermes speratus*, *P. apteru*s and *L. migratoria*^24,25,27–30,32,33,36,38,40,66,67^.

**Figure 4:**
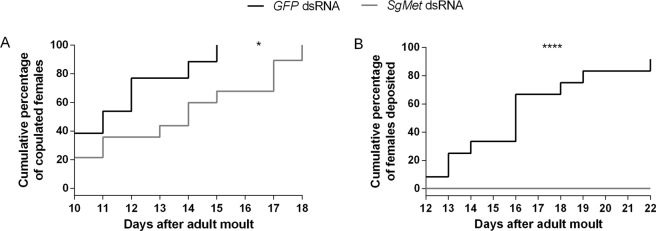
Copulation behaviour and oviposition of *dsSgMet* injected locusts. Copulation behaviour and post-copulation effects were observed from 12 individuals per condition starting on day 8 of the adult stage. (**A**) The cumulative percentage of copulating females (actual connection between male and female genitalia) and (**B**) the cumulative percentage of eggs deposited by the females are showed. Statistically significant differences (p) between the two conditions were found via a log-rank (Mantel-Cox) test and are indicated by asterisks (*p< 0.05; ****p< 0.0001).

Met knockdown in female bed bugs (*C. lectularius*) also caused a reduced number of eggs deposited^27^. In our study, an effect on copulation behaviour as well as on oviposition was witnessed. Where control females all copulated on day 15, a delay was seen in the *dsSgMet* injected individuals (Fig. 4A). Moreover, none of these experimental locusts was capable of depositing their eggs within the set experimental timeframe (Fig. 4B), likely the result of incomplete oocyte maturation (Fig. 3J) and no increase in survival was observed in any of the knockdown experiments.

### *Sg*Met is also crucial for ovarian ecdysteroid biosynthesis

**Figure 5:**
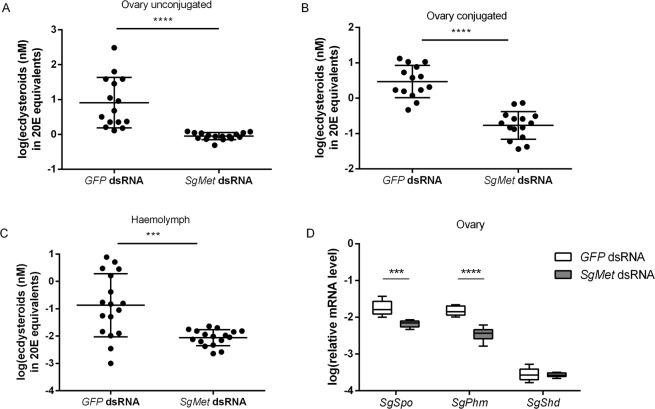
Ecdysteroid levels and expression of *Halloween* genes upon silencing of *SgMet* in adult female locusts. Ecdysteroid levels (20E equivalents in nM) as well as *Halloween* gene expression were measured in 12-day-old female control and experimental locusts; (**A**) non-conjugated ovarian ecdysteroid levels, (**B**) conjugated ovarian ecdysteroid levels and (**C**) haemolymph ecdysteroid levels. Each point (log transformed) represents a measurement (mean ± S.E.M). (**D**) Relative expression levels of three *Halloween* genes, *i.e. SgSpo, SgPhm* and *SgShd* were measured in the ovaries of 12-day-old *dsSgMet* injected female locusts. The data (log transformed) are shown as box plots (min to max) of four independent pools of five animals, run in duplicate and normalized to *β-actin* and *EF1α* transcript levels. (**A**–**D**) Statistically significant differences (p) between the measurements were found, after a log transformation, via a t-test (with or without two-sided Welch’s correction) and indicated by asterisks (***p < 0.001, ****p < 0.0001).

**Figure 6:**
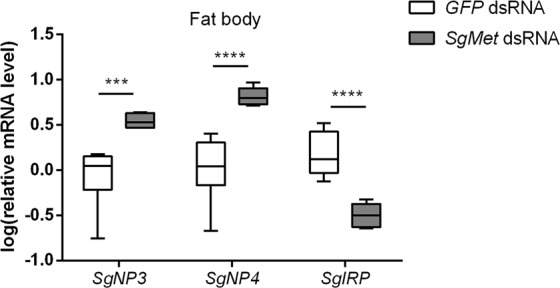
JH receptor knockdown alters the levels of neuroparsin and insulin-related peptide precursor transcripts. Relative *SgNP3*, *SgNP4* and *SgIRP* transcript levels were measured in the fat body of 12-day-old *dsSgMet* injected female locusts. The data (log transformed) are shown as box plots (min to max) of four independent pools of five animals, run in duplicate and normalized to *β-actin* and *EF1α* transcript levels. Statistically significant differences (p) between the measurements were found, after a log transformation, via a t-test (with or without two-sided Welch’s correction) and are indicated by asterisks (***p < 0.001; ****p < 0.0001).

In vitellogenic female locusts, the developing basal oocytes also incorporate ‘free’ and conjugated ecdysteroids, while a rise in the circulating ecdysteroid levels can be observed in the haemolymph, as described previously^68,69^. The majority of the ecdysteroids produced by the follicle cell layer are incorporated in the growing oocytes as conjugates. Only a small fraction is stored in the oocytes as ‘free’ ecdysteroids. An enzyme immunoassay (EIA) was used to determine the effect of *SgMet* knockdown on ecdysteroid levels in the developing oocytes and the haemolymph. Silencing *SgMet* resulted in a significantly reduced level of both circulating and ovarian ecdysteroids (expressed in 20E equivalents) compared to the control locusts (Fig. 5A–C). Ecdysteroid biosynthesis in adult locusts mainly takes place in the follicle cell layer of the developing oocytes^70,71^ where the steroid hormone precursor cholesterol is converted to 20E in a series of enzymatic reactions involving the *Halloween* genes, first characterized in *D. melanogaster*^72^. Three of these *Halloween* genes, *i.e. Spook* (*Spo*), *Phantom* (*Phm*) and *Shade* (*Shd*), encoding cytochrome P450 enzymes were already functionally characterized in *S. gregaria*^68,73^. *SgSpo*, encoding an enzyme acting in the Black Box of the ecdysteroid biosynthetic pathway, is considered to catalyse the rate-limiting step. *SgShd* is important in the conversion of ecdysone to the active hormone, 20E. In adult locusts, relative mRNA levels of all three are highest in the ovaries and degenerating PGs. The reduced level of circulating and ovarian ecdysteroids in the *dsSgMet* treated animals is also evidenced in the ovarian transcript levels of both *SgSpo* and *SgPhm* (Fig. 5D). Our data indicate that the initiation of ecdysteroid biosynthesis by the reproductive organs is situated downstream of the JH-mediated initiation of oocyte growth and vitellogenesis. The start of ecdysteroidogenesis in locusts may be directed by a nutritional state-dependent factor binding to the Venus Kinase Receptor (VKR), a receptor tyrosine kinase as evidenced by a recent study by Lenaerts *et al*.^74^. Knockdown of *SgVKR* in adult females results in reduced oocyte size, ovarian and circulating ecdysteroid levels. Relative transcript levels of several *Halloween* genes were negatively affected^74^. Downregulation of *SgMet* resulted in a significant reduction of relative *SgVKR* transcript levels in the ovary (Supplementary Fig. S4A). In the mosquito, *A. aegypti*, the ligand for this receptor is the Ovarian Ecdysteroidogenic Hormone (OEH), an 83 AA long peptide released from medial neurosecretory cells in the mosquito brain upon blood feeding which induces the ovaries to produce ecdysteroids. These ecdysteroids, as in other higher insects, will stimulate the fat body to produce yolk protein^75,76^. Interestingly, OEH is considered as a member of the neuroparsin family, which possesses a typical, conserved pattern of Cys residues^48^. Most insects seem to encode a single NP gene, while in some species, such as *D. melanogaster*, no NP has been discovered^45^. However, in *S. gregaria* four *SgNP* precursor transcripts are found, for which transcript profiling studies were performed in the past^77,78^. In gregarious locusts, transcripts for *SgNP1* and *SgNP2* are predominantly detected in the brain; whereas *SgNP3* and *SgNP4* are also detected in female fat body and ovaries. As previous research from our lab and current data in Supplementary Fig. S5A,B show, transcripts of *SgNP3* and *SgNP4* are highly regulated during the first gonadotrophic cycle, suggesting these peptides play a role in reproductive physiology. A decline of both *SgNP3* and *SgNP4* transcripts was observed approximately at the time when *SgVg* transcript levels started rising in the fat body. In the *dsMet* treated animals the relative mRNA levels of both *SgNP3* and *SgNP4* are higher compared to the control animals in fat body, while the relative transcript level of *SgNP4* is also increased in the ovary (see Fig. 6 and Supplementary Fig. S4B). At present, it is still unclear whether the changes in *SgNP3* and *SgNP4* expression are caused by changes in JH signalling or to indirect effects via other regulatory pathways that have been affected in the *dsMet* treated locusts. JH injection into the haemocoel of day 5 adult female locusts results in an increase of *SgNP3* and *SgNP4* transcript levels in both female fat body and ovary^79^. In our current study, we observe that *SgMet* expression in the ovary is not significantly influenced by the systemic knockdown, which could be due to weaker RNAi sensitivity^57^ and/or stronger compensatory effects in this tissue. In the CA, we observe another kind of compensatory effect resulting in significantly increased expression of JH biosynthetic enzymes. So, it could be that these compensatory effects may also have resulted in increased ovarian *SgNP4* levels. However, this does not explain why both *SgNP3* and *SgNP4* transcript levels are strongly increased in the fat body, since in this tissue knockdown of *SgMet* and reduced JH signalling are clearly evidenced by significantly reduced transcript levels of *SgMet* and *SgKrh1*, respectively. Another receptor that may be involved in causing the observed changes in *SgNP* transcript levels is *Sg*VKR. In the current study, we show that *SgVKR* transcript levels are significantly reduced in *dsMet* treated desert locusts. The observed effect on *SgNP* transcript levels might be due to these reduced *SgVKR* levels, since Lenaerts *et al*.^74^ have shown that knockdown of *SgVKR* resulted in increased *SgNP3* and *SgNP4* levels in locusts. Nevertheless, the occurrence of other currently unknown mechanisms cannot be ruled out.

### Knockdown of JH receptor Met alters expression of insulin-related peptide and neuroparsin precursor genes in an opposite way

Previous research has shown that IRP has a gonadotropic role, while NPs have an anti-gonadotropic role in the female reproductive physiology of locusts. Studies in *S. gregaria* revealed that an increase in *SgIRP* transcript levels can be observed during specific reproductive events such as vitellogenesis and intensive growth of the oocytes^80^. The effect of this peptide was later evidenced in an RNAi-mediated study in *S. gregaria* by Badisco *et al*.^47^ showing lower *SgVg* transcript levels and smaller oocytes compared to control animals upon ds*IRP* injections^47^. Playing a vital role as nutritional sensor, the ISP has been described in different insect species as having a gonadotropic effect. In dipterans, insulin-like peptides (ILPs) are involved in stimulating egg production and affecting germ line cyst development rate as well as progression through vitellogenesis^81–83^. In the yellow fever mosquito, *A. aegypti*, ILP3 stimulates yolk uptake by the developing oocytes^81^. Moreover, insulin signalling is also required to induce vitellogenin production in the fat body^84^ and to stimulate ecdysteroid production in the ovaries^85^. Furthermore, *D. melanogaster* female ISP mutants generally show reduced vitellogenesis^86^. In addition, mutations in the insulin receptor result in a decrease of JH synthesis suggesting the involvement of the ISP in the regulation of JH biosynthesis^87,88^. In the red flour beetle, *T. castaneum*, it was shown that JH is able to regulate ILP synthesis, which in turn regulates Vg synthesis^37^. In the adult female beetles, both JH and feeding are crucial in the onset of ILP production in the fat body and brain after which ILPs will trigger the Vg synthesis through removal of a Vg transcriptional repressor^36,37,89^. RNAi of the insulin receptor in *B. germanica* adult females resulted in a reduction in JH biosynthesis, *Vg* expression and ovarian development^90^. On the other hand, silencing of *SgNP*s in the RNAi-mediated study by Badisco *et al*.^47^ resulted in higher *SgVg* transcript levels and bigger oocytes in 12-day-old female locusts^47^. This anti-gonadotropic action of NPs has also been proven in *L. migratoria*^80^.

Our current results show that during the locust’s first gonadotrophic cycle, a decrease in relative *SgNP3* and *SgNP4* transcript levels coincides with an increase in relative *SgIRP* expression in the locust fat body (Supplementary Fig. S5). Also in the *dsSgMet* treated females, a significant increase in *SgNP*3 and *SgNP4* expression was measured in the fat body. *SgIRP* expression on the other hand decreased (Fig. 6). This clearly indicates a consistent cross-talk between *SgNP* and *SgIRP* signalling downstream of JH.

**Figure 7:**
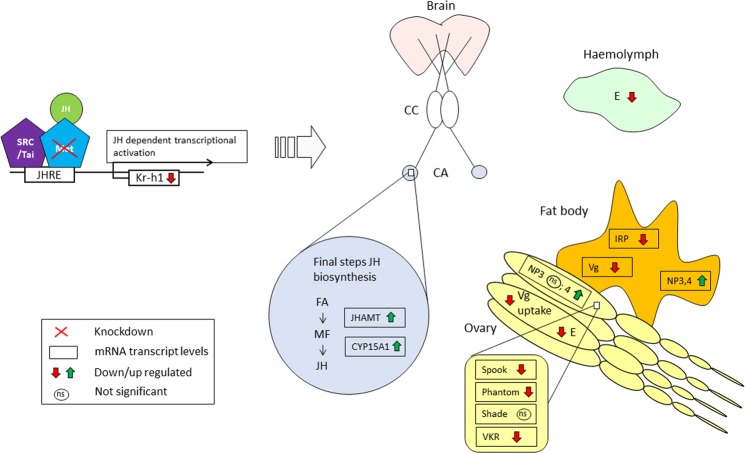
A schematic of the different effects upon *SgMet* silencing. All the effects observed upon *SgMet* silencing are summarized. Effects on the relative mRNA transcript levels are shown in rectangular boxes with arrowheads indicating down or up regulation upon *SgMet* knockdown. CA = Corpora allata; CC = Corpora cardiaca; CYP15A1 = Cytochrome P450 enzyme 15A1; FA = Farnesoic acid; E = Ecdysteroid; IRP = Insulin-related peptide; JH = Juvenile hormone; JHAMT = Juvenile hormone acid methyl transferase; Kr-h1 = Krüppel-homolog 1; Met = Methoprene-tolerant; MF = methylfarnesoate; NP = Neuroparsin; ns = not significant; SRC/Tai = Steroid receptor coactivator/Taiman; Vg = Vitellogenin.

All the above mentioned effects observed upon *SgMet* silencing are summarized in Fig. 7.

## Conclusions

Our results show that the knockdown of *SgMet* in female locusts results in a failure to initiate vitellogenin expression in the fat body as well as oocyte growth in the ovaries. These *dsSgMet* treated animals are still able to copulate, albeit later, but they do not oviposit. As such, strategies ensuring a specific knockdown or inhibition of components of the JH signalling pathway, or of functionally linked pathways, may prove to be excellent methods for specifically targeting locust reproduction and associated swarm formation. Our study also indicates that upon *SgMet* knockdown some *SgNP* transcripts are significantly upregulated in the locust fat body, while the *SgIRP* levels are downregulated. In fact, these observations appear to be very well in line with their previously reported opposing roles in the control of reproduction. Furthermore, we show that *SgMet* knockdown strongly influences the expression of JH biosynthesis enzymes in the CA, as well as of ecdysteroid biosynthesis enzymes in the ovary, as evidenced by lower ovarian concentrations of both free and conjugated ecdysteroids. In conclusion, our study sheds more light on the essential role of the JH receptor *SgMet*, as well as on the interdependencies between different endocrine pathways involved in the control of reproduction in female desert locusts.

## Materials and Methods

### Rearing of animals

The desert locusts, *S. gregaria*, were reared under crowded conditions as previously described by Lenaerts *et al*.^74^. Sand/turf pots, into which eggs were deposited by mated females, were set apart once a week in clean cages.

### Tissue collection

The tissues of interest were dissected as previously described by Lenaerts *et al*.^74^. For the tissue and temporal expression profile of the genes of interest, tissues were collected in three pools of ten animals each. Tissues for the RNAi experiment were collected in four pools consisting of five animals each. The collected tissues were used in an RNA extraction protocol or stored at −80 °C until further processing.

### RNA extraction and cDNA synthesis

Fat body, ovaries, muscles and midgut of female adult locusts as well as the male reproductive system (testes + AGs) were pooled in MagNA Lyser Green Beads Tubes (Roche) and homogenized using a MagNA Lyser instrument (20 s, 5000 rpm; Roche). Subsequently, total RNA was extracted using the RNeasy Lipid Tissue Kit (Qiagen) according to the manufacturer’s protocol. To prevent genomic DNA contamination, an on-column DNase digestion (RNase-free DNase set, Qiagen) was performed. Total RNA of the small sized tissues (CA/CC complex, PGs and suboesophageal ganglion (SOG)) was extracted using the RNAqueous-Micro Kit (Ambion) according to the manufacturer’s protocol, including the optional DNase step. Quality and concentration of the resulting RNA samples was measured using a Nanodrop spectrophotometer (Nanodrop ND-1000, Thermo Fisher Scientific Inc.). For each RNA sample, equal amounts of RNA (1000 ng for brain, fat body, ovaries, muscles, midgut, testes and AGs and 200 ng for PGs, CA/CC complex and SOG) were transcribed in subsequent cDNA synthesis using a mix of random hexamers and oligo(dT) primers according to the manufacturer’s protocol (PrimeScript^TM^ RT Reagent Kit, TaKaRa, Invitrogen Life Technologies). The obtained cDNA was diluted ten-fold with Milli-Q water (Millipore).

### Quantitative real-time PCR

Prior to quantitative real-time PCR (qRT-PCR) transcript profiling, optimal housekeeping genes were selected using geNorm software^91^. Therefore, several previously described housekeeping genes^92^ were tested for their stability in the designed experiment and *β-actin* and *EF1α* appeared to be most stable in the studied samples. Primer Express software (Applied Biosystems) was used to design qRT-PCR primers for the different target genes (Supplementary Table S1). Subsequent primer validation and qRT-PCR reactions were performed as previously described by Lenaerts *et al*.^74^.

All qRT-PCR results were normalized to both the *β-actin* and *EF1α* transcript levels and calculated relative to the transcript level in a calibrator sample according to the comparative Ct method (ΔΔCt)^91^. qRT-PCR was used to determine the tissue and temporal distributions of the genes of interest in the adult stage. Therefore, cDNA samples were used from adult female locusts, except for the male AGs and testes. Moreover, statistically significant differences between the *dsSgMet* injected and control adult female locusts were found via a t-test on the log transformed data (with or without two-sided Welch’s correction) using GraphPad Prism 6 (GraphPad Software Inc.). Correlation coefficients between temporal expression profiles of the genes of interest were found via a Pearson correlation calculation using GraphPad Prism 6 (GraphPad Software Inc.).

### RNA interference experiments

DsRNA constructs were prepared using Ambion’s MEGAScript RNAi kit following the manufacturer’s protocol and as previously described by Lenaerts *et al*.^74^. In short, a linear transcript with 5′ T7 promotor sequences was used in a high yield RNA transcription reaction. Linear transcripts for the targets of interest were PCR amplified using REDTaq DNA polymerase (Sigma), *S. gregaria* fat body cDNA and the primers given in Supplementary Table S2. The amplicons were sequenced to confirm target specificity and used in a high-yield RNA transcription reaction using T7 RNA polymerase. ssRNA and DNA were further removed in a nuclease digestion step. The resulting dsRNA was analysed on gel and using the Nanodrop spectrophotometer for integrity, quality and concentration.

Newly moulted adult female locusts, synchronized on the day of ecdysis, were injected with 8 µl dsRNA against *SgMet* (500 ng dsRNA/locust, diluted in *S. gregaria* ringer). A second and third boost injection was given on days four and eight after final moult to ensure a lasting knockdown of target gene mRNA levels. A second group of control locusts was injected with dsRNA against *GFP* following the same injection scheme. From previous observations of our *S. gregaria* colony, it is known that female adults are fully in the vitellogenic stage of oocyte maturation 12 days after their final moult. Therefore, all the tissues and samples of interest were dissected and collected on this day. In a follow-up experiment, the same injection scheme was followed and copulation behaviour and oviposition were observed. Starting on day 8, the female locusts were transferred to individual cages, supplied with oats and fed daily with fresh cabbage. On day 10 of the females’ adult life, one sexually mature virgin male was introduced into the separate cages and mating behaviour and copulation (actual connection between male and female genitalia) was registered. If no copulation was observed within two hours of the male’s introduction, the male was removed and another mating was attempted the following day with another virgin male. After mating, pots filled with a humid sand/turf mixture were supplied to the females to allow oviposition. These pots were checked daily for egg pods. Statistically significant differences between the two conditions were found via a log-rank (Mantel-Cox) test using GraphPad Prism 6 (GraphPad Software Inc.).

### Measurement of oocyte length

Terminal oocytes, *i.e*. oocytes at the base of the ovarioles, were carefully removed from the dissected ovaries and their length was measured using millimetre graph paper. Statistically significant differences between the *dsSgMet* injected and control adult female locusts were found via a nonparametric Mann-Whitney test using GraphPad Prism 6 (GraphPad Software Inc.).

### Microscopy and histological analysis

Images of the dissected ovaries and ovarioles as well as of the oocyte sections of *dsSgMet* and *dsGFP* (*GFP* dsRNA) treated animals were made as previously described by Lenaerts *et al*.^74^.

### Ecdysteroid measurements using an enzyme immunoassay

Female locusts were pierced behind the hind leg on the dorsal side allowing haemolymph to flow out. 10 µl of haemolymph was collected from each adult female using a glass capillary tube, immediately transferred to 90 µl of 100% ethanol and stored at −20 °C until further processing as described by Marchal *et al*.^68^.

One ovarium of each female locust was dissected and transferred to 1 ml of 100% ethanol after which it was stored at −20 °C until further processing. These ovarium samples were homogenized using a bar sonicator and subsequently for 10 min at 60 °C. After centrifugation at 10 000 g (10 min), the supernatants were transferred to a fresh Eppendorf tube and the remaining pellets were re-extracted twice by adding 1 ml of 100% ethanol. Combined extracts were dried in a SpeedVac concentrator and redissolved in 1 ml of 70% ethanol and 1 ml of 100% hexane in order to remove the apolar lipids and as such obtain optimal EIA measurements. Both ‘free’ and total ecdysteroid levels were determined where the ‘free’ ecdysteroids are non-conjugated. The total ecdysteroid levels were determined after enzymatic conversion of the conjugated ecdysteroids into ‘free’ ecdysteroids.

Ecdysteroid titers in *S. gregaria* haemolymph and ovaries were measured using an EIA, modified from Porcheron *et al*.^93^ and discussed by Pascual *et al*.^94^ and Lafont *et al*.^93–95^. In brief a peroxidase conjugate of 20E is used as tracer together with rabbit L2 polyclonal antibodies against ecdysteroids. Both serum and tracer were a kind gift by Prof. J.P. Delbecque (Université de Bordeaux, France).

The resulting absorbance values were compared to the calibration curve obtained with 20E as a standard. Since the data were normally distributed upon log transformation, a t-test (with or without two-sided Welch’s correction) was performed using GraphPad Prism 6 (GraphPad Software Inc.) to test for statistically significant differences between the data.

## Supplementary information

Supplementary Figures + Tables

## Data Availability

All data generated or analysed during this study are included in this published article (and its Supplementary Information files).
