# Supplementary material for: Juvenile Hormone receptor Met is essential for ovarian maturation in the Desert Locust, Schistocerca gregaria
Source: Sci Rep. 2019 Jul 25;9:10797. doi: 10.1038/s41598-019-47253-x (PMC6658565; doi:10.1038/s41598-019-47253-x)
Supplement: Supplementary file 1 — Supplementary Figures + Tables [file 41598_2019_47253_MOESM1_ESM.docx]

**Juvenile Hormone receptor Met is essential for ovarian maturation in the Desert Locust, *Schistocerca gregaria***

Marijke Gijbels, Cynthia Lenaerts, Jozef Vanden Broeck^*^, Elisabeth Marchal^§,*^

Research group of Molecular Developmental Physiology and Signal Transduction, KU Leuven, Zoological Institute, Naamsestraat 59 box 2465, 3000 Leuven, Belgium

**^§^**Current address: Imec, Kapeldreef 75, B- 3001 Leuven, Belgium

**^*^Correspondence:**

Elisabeth Marchal: [elisabeth.marchal@imec.be](mailto:elisabeth.marchal@imec.be)

Jozef Vanden Broeck: [jozef.vandenbroeck@kuleuven.be](mailto:jozef.vandenbroeck@kuleuven.be)

[Marijke.Gijbels@kuleuven.be](mailto:Marijke.Gijbels@kuleuven.be)

[Cynthia.Lenaerts@kuleuven.be](mailto:Cynthia.Lenaerts@kuleuven.be)

[Jozef.VandenBroeck@kuleuven.be](mailto:Jozef.VandenBroeck@kuleuven.be)

[Elisabeth.Marchal@imec.be](mailto:Elisabeth.Marchal@imec.be)





**Supplementary Figure S1**: **Tissue distribution profile** **of the relative transcript levels of *SgMet***. Tissues were dissected from 10-day-old adult female locusts, except for the male gonads and accessory glands. The data represent mean ± S.E.M. of three independent pools of ten animals, run in duplicate and normalized to *β-actin* and *EF1α* transcript levels. Abbreviations X-axis: Br = Brain; CA = Corpora allata; PG = Prothoracic glands; SOG = Suboesophageal ganglion; MG = Midgut; Fb = Fat body; Ov = Ovaries; AG = Accessory glands.





**Supplementary Figure S2:** **Knockdown efficiency of *SgMet* dsRNA injections.** The knockdown efficiency of *SgMet* dsRNA injections was assessed in three different tissues on day 12 after adult eclosion, *i.e.* CA/CC complex, fat body and ovaries. The data (log transformed) are shown as box plots (min to max) of four independent pools of five animals, run in duplicate and normalized to *β-actin* and *EF1α* transcript levels. Statistical significant differences between the measurements were found via a t-test (with or without two-sided Welch’s correction) and are indicated by asterisks (** = p < 0.01; **** = p < 0.0001).


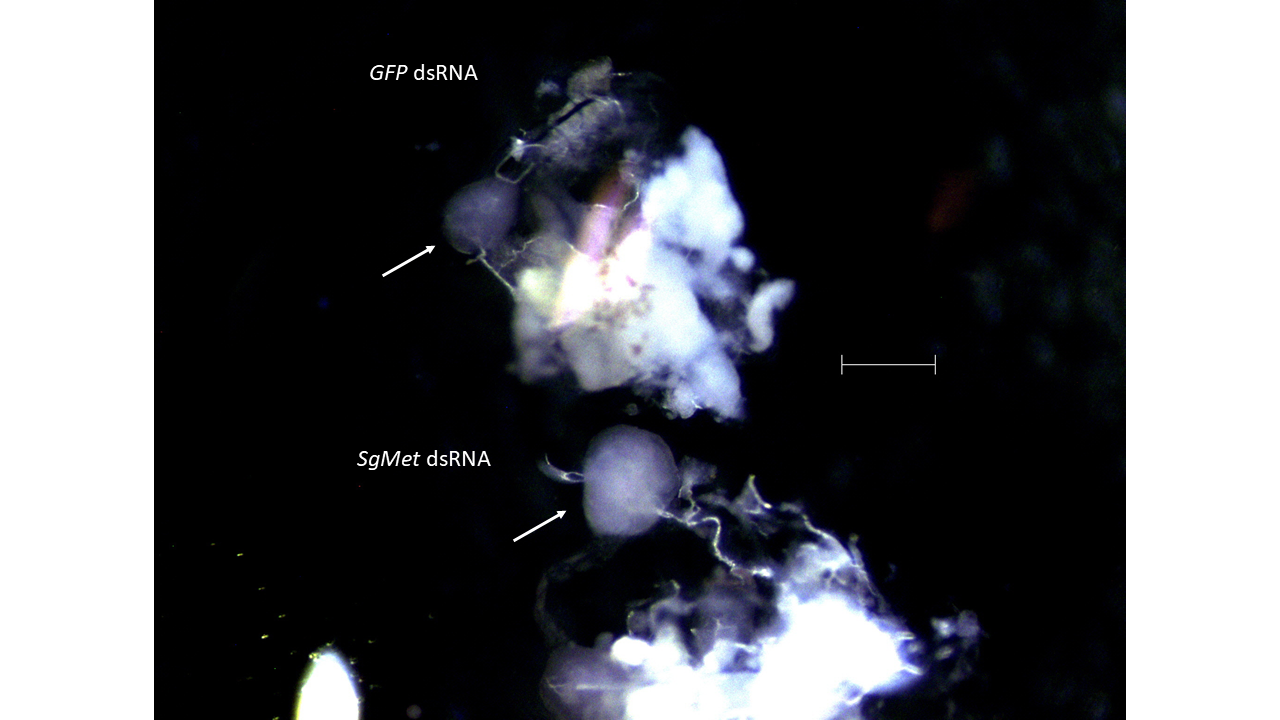


**Supplementary Figure S3**: **Comparing gland sizes of dissected CA between control and experimental adult female locusts.** The CA/CC complex of 12-day-old control and *SgMet* dsRNA injected female locusts was dissected where after CA gland sizes (indicated by an arrow) were compared. Scale bar = 1 mm.






**Supplementary Figure S4:** ***SgVKR*, *SgNP3* and *SgNP4* relative transcript levels.** Relative expression level of (A) *SgVKR* and (B) *SgNP3* + *SgNP4* was measured in the ovaries of 12-day-old *SgMet* dsRNA treated locusts and control locusts. The data (log transformed) are shown as box plots (min to max) of four independent pools of five animals, run in duplicate and normalized to *β-actin* and *EF1α* transcript levels. Statistical significant differences (p) between the measurements were found, after a log transformation, via a t-test (with or without two-sided Welch’s correction) and are indicated by asterisks (** = p < 0.01).

**Supplementary Figure S5: Relative transcript levels of *SgNP3*, *SgNP4* and *SgIRP* throughout the first gonadotrophic cycle**. (A) *SgNP3*, (B) *SgNP4* and (C) *SgIRP* relative transcript levels were determined in the fat body of freshly moulted adult females (day 0 until day 18). *SgVg1* expression is included as well. Samples were collected every other day during the first gonadotrophic cycle. The data represent mean ± S.E.M. of three independent pools of ten animals, run in duplicate and normalized to *β-actin* and *EF1α* transcript levels.

**Supplementary Table S1: Oligonucleotide sequences of primers used for qRT-PCR.**

| **Reference/**  **target genes** | **Forward primer** | **Reverse primer** |
| --- | --- | --- |
| *SgAct* | 5’- AATTACCATTGGTAACGAGCGATT -3’ | 5’- TGCTTCCATACCCAGGAATGA -3’ |
| *SgEF1α* | 5’- GATGCTCCAGGCCACAGAGA -3’ | 5’- TGCACAGTCGGCCTGTGAT -3’ |
| *SgMet* | 5’- GGTGCCTGAAGAGGAAGAAA -3’ | 5’- ATGGAGGTGATGAAGGAGAAAG -3’ |
| *SgKr-h1* | 5’- CTCCAAGACGTTCATCCAGAG -3’ | 5’- TGCTTGGAGCAGGTGAAG -3’ |
| *SgIRP* | 5’- CCGTGGCAACTACAACACCAT -3’ | 5’- TCCGCGTCCGACACATCT -3’ |
| *SgNP3* | 5’- GCGGAATCGGCATGGA -3’ | 5’- TCACAGAGCAACCGGAACATT -3’ |
| *SgNP4* | 5’- TGGCGACTCTCCAGTGCTT -3’ | 5’- TGACACATTCATTCCTCTTCTGACA -3’ |
| *SgVg1* | 5’- CCGCTGAACATCACTGCAAT -3’ | 5’- ACTTGGGCCAAATGGATGAG -3’ |
| *SgVg2* | 5’- GCTACCCGCAATCTGTAAAATACA -3’ | 5’- CGACTGTGAAAGGGCATTGA -3’ |
| *SgCYP15A1* | 5’- AAAGCAACTTCATCATTCACAGATG -3’ | 5’- CAGAGCCAGCCATGAACAAA -3’ |
| *SgJHAMT* | 5’- CGGAGCAAAGGCAAGCA -3’ | 5’- CCACTTCACCGCCTGGTTT -3’ |
| *SgSpo* | 5’- CAACATCTTCACCAGCTACATGTG -3’ | 5’- GGGTCGTCGTAGTCGAAGGA -3’ |
| *SgPhm* | 5’- CGCAGAGCCCGGACAAC -3’ | 5’- CGAACATGTCGGCCATGA -3’ |
| *SgShd* | 5’- CCGCCGTCATTGACTTCATA -3’ | 5’- GTGAGCTCCCAAGCGTGG -3’ |
| *SgVKR* | 5’- GCATCTTGGCATTGATTTGCTA -3’ | 5’- GGAATCTCCCATTTGTCAAGAGTT -3’ |

Abbreviations: *Sg* = *Schistocerca gregaria*, *Act* = *β-actin*; *EF1α* = *Elongation factor 1α*; *Met* = *Methoprene-tolerant*; *Kr-h1* = *Krüppel-homolog 1*; *IRP* = *Insulin-related peptide*; *NP* = *Neuroparsin*; *Vg* = *Vitellogenin*; *CYP15A1* = C*ytochrome P450 enzyme 15A1 (methyl farnesoate epoxidase)*; *JHAMT* = *Juvenile hormone acid methyltransferase*; *Spo* = *Spook*; *Phm* = *Phantom*; *Shd* = *Shade*; *VKR* = *Venus Kinase Receptor.*

**Supplementary Table S2:** **Oligonucleotide sequences of primers used for dsRNA synthesis.** Bases indicated in ‘bold’ represent the T7 promotor sequences used in the preparation of dsRNA constructs.

| RNAi constructs | F-primer | R-primer |
| --- | --- | --- |
| *SgMet* | 5’- **TAATACGACTCACTATAGGGAGA**  AATGAGCCGTTTGGCAGTTCCAC -3’ | 5’- **TAATACGACTCACTATAGGGAGA**  GGCGGTGCAGCACTCATAGC -3’ |
| *SgMet2* | 5’- **TAATACGACTCACTATAGGGAGA**  GCTATGAGTGCTGCACCGCC -3’ | 5’- **TAATACGACTCACTATAGGGAGA**  AAGAGGCAGGCCACTAGGTT -3’ |
| *GFP* | 5’-**TAATACGACTCACTATAGGGAGA**  AAGGTGATGCTACATACGGAA -3’ | 5’-**TAATACGACTCACTATAGGGAGA** ATCCCAGCAGCAGTTACAAAC-3’ |

Abbreviations: *Sg* = *Schistocerca gregaria*, *Met* = *Methoprene-tolerant*; *GFP = Green fluorescent peptide.*
